# An Incidental Embolism

**DOI:** 10.1016/j.acepjo.2025.100216

**Published:** 2025-06-27

**Authors:** Emma Alley, Eric Melnychuk

**Affiliations:** 1 Department of Emergency Medicine, Geisinger Medical Center, Danville, Pennsylvania, USA; 2 Critical Care Medicine, Geisinger Medical Center, Danville, Pennsylvania, USA

**Keywords:** central venous catheter, foreign body

## 1 Case Presentation

A 70-year-old man presented to the emergency department for increased falls and generalized weakness at home. He had a medical history of myocardial infarction, coronary artery bypass grafting, end-stage renal disease on hemodialysis, and a ruptured aneurysm of his left arteriovenous dialysis fistula. He also had a history of multiple tunneled dialysis catheters placed, the most recent being 7 months prior. Computed tomography of the chest was obtained as part of the workup.

## 2 Diagnosis

Computed tomography of the chest revealed an incidental intravascular retained catheter fragment located in his right pulmonary artery (Fig). Interventional radiology was consulted for the evaluation and removal of the foreign body. Retained catheter fragments from venous access can result in complications including arrythmias, infection, and death.^1^ In our case, a chest x-ray from 1 month prior did not show evidence of a retained foreign body despite his most recent intravascular procedure occurring 7 months prior. Up to 11.6% of patients living with retained foreign bodies are asymptomatic and have been identified incidentally.^1^ Percutaneous approaches to intravascular foreign bodies appear to be the primary method of removal.^2^^,^^3^ This case highlights the importance of examining guidewires and catheters placed intravascularly to ensure that they are intact before placement and after removal, as this can reduce delayed adverse procedural outcomes.

**Figure:**
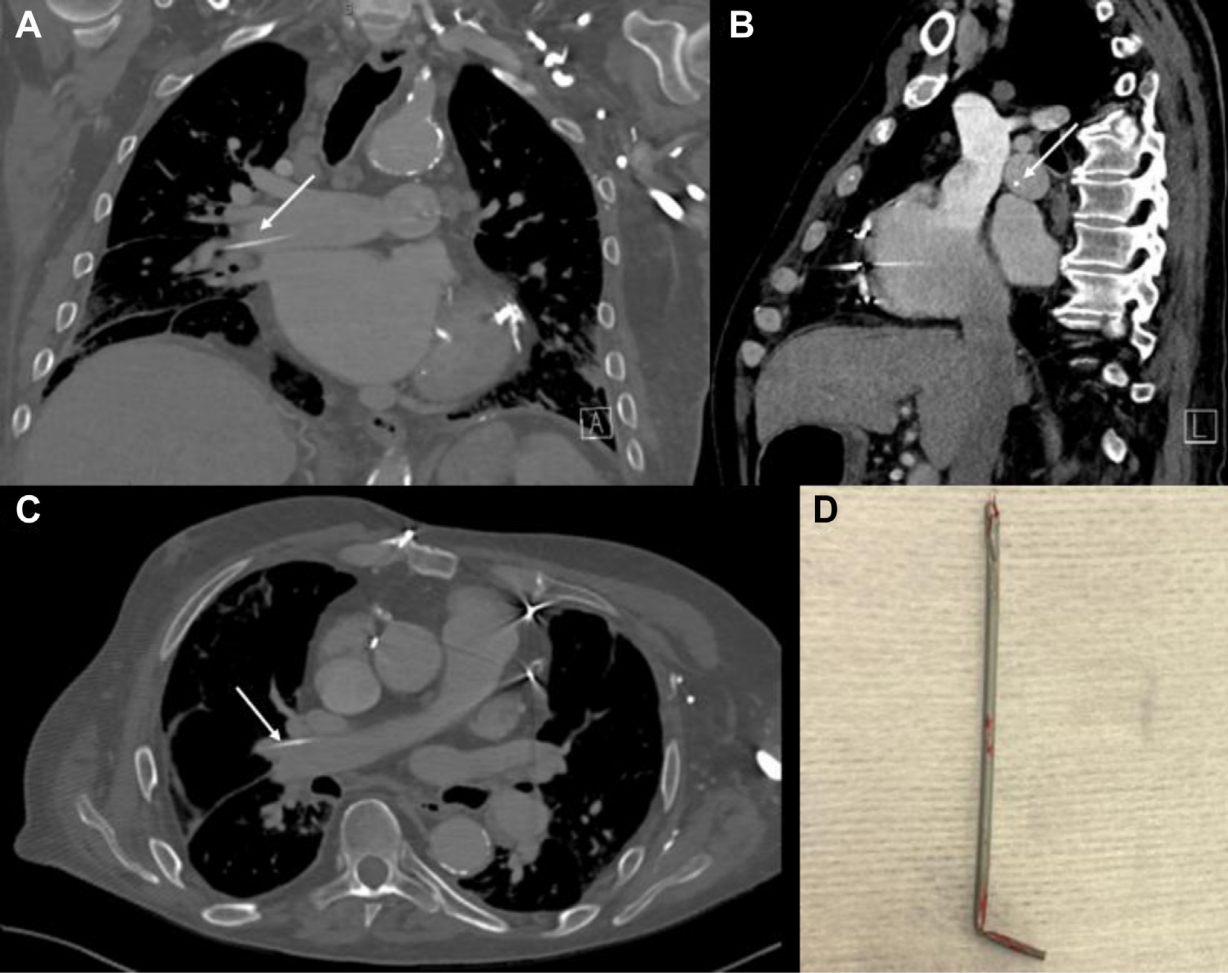
The image shows multiple views of a retained catheter in the right pulmonary artery extending into the right middle lobar artery. Arrows point to the retained catheter. (A) Coronal view. (B) Sagittal view. (C) Transverse view. (D) Image of the catheter after retrieval by Interventional Radiology.

## Funding and Support

By *JACEP Open* policy, all authors are required to disclose any and all commercial, financial, and other relationships in any way related to the subject of this article as per ICMJE conflict of interest guidelines (see www.icmje.org). The authors have stated that no such relationships exist.
